# The role of quality of care and attitude towards disability in the relationship between severity of disability and quality of life: findings from a cross-sectional survey among people with physical disability in China

**DOI:** 10.1186/1477-7525-12-25

**Published:** 2014-02-23

**Authors:** Qiao-Lan Zheng, Qi Tian, Chun Hao, Jing Gu, Ramona Lucas-Carrasco, Jian-Ting Tao, Zuo-Yi Liang, Xin-Lin Chen, Ji-Qian Fang, Jian-Hua Ruan, Qiu-Xiang Ai, Yuan-Tao Hao

**Affiliations:** 1 Department of Medical Statistics and Epidemiology & Center for Health Informatics Research & Guangdong Key Laboratory of Medicine, Laboratory of Health Informatics, School of Public Health, Sun Yat-sen University, Guangzhou Guangdong, P.R. China; 2 Department of Statistics, Guangzhou Health Information Center, Guangzhou, Guangdong, P.R. China; 3 Takemi Program in International Health, Department of Global Health and Population, Harvard School of Public Health, Boston, Massachusetts, USA; 4 Department of Methodology and Behavioral Sciences, University of Barcelona & SGR 822 Generalitat de Catalunya, Barcelona, Spain; 5 Cambridgeshire and Peterborough NHS Foundation Trust, Cambridge, UK; 6 Guangzhou Service Center of Assistive Devices, Guangzhou, Guangdong, P.R. China; 7 Guangzhou Disabled Persons’ Federation, Guangzhou, Guangdong, P.R. China; 8 Department of Preventive Medicine and Health Statistics, College of Fundamental Medical Science, Guangzhou University of Chinese Medicine, Guangzhou, Guangdong, P.R. China

**Keywords:** Quality of life, Quality of care and support, Attitude towards disability, Severity of disability, People with physical disability, Structural equation modeling

## Abstract

**Background:**

People with physical disability (PWPD) is the largest subgroup of people with disability (PWD) in China, but few studies have been conducted among this vulnerable population. The objective of this study was to investigate the level of quality of life (QoL), self-perceived quality of care and support (QOCS), severity of disability and personal attitude towards disability among people with physical disability in China, as well as to identify how QoL can be affected by severity of disability through QOCS and personal attitude towards disability among PWPD.

**Methods:**

A cross-sectional study was conducted among 1,853 PWPD in Guangzhou, China. Data were collected on participants’ QoL, QOCS, personal attitude towards disability and severity of disability. Structural equation modeling was used to examine the effects of the other variables on QoL.

**Results:**

Even with a mild disability (mean score:1.72), relatively low levels of QoL (mean score: 2.65- 3.22) and QOCS (mean score: 2.95 to 3.28), as well as unfavorable personal attitude towards disability (mean score: 2.75 to 3.36) were identified among PWPD. According to SEM, we found that the influence of severity of physical disability on QoL is not only exerted directly, but is also indirectly through QOCS and their personal attitudes towards disability, with QOCS playing a more important mediating role than PWPD’s attitudes towards their own disability.

**Conclusions:**

Unfavorable health status was identified among PWPD in China. Focusing on improvement of assistance and care services has the potential to substantially improve PWPD’s QoL. Further research should focus on understanding the needs and their current state of health care of PWPD in China thus being able to develop better interventions for them.

## Background

Disabilities have caused a substantial global disease burden [1]. By the end of 2010, there were approximately 85 million people living with disability in China [2]. Compared to other types of disability (visual, hearing and speech, intellectual, and mental), people with physical disability (PWPD) account for 30% of people with disability (PWD) in China, constituting the largest subgroup of disability [2]. Few studies have been conducted among PWD in China, especially among PWPD. Existing disability-related studies in China mostly focused on people living with intellectual disability [3], who confront substantially different barriers from PWPD. World Health Organization (WHO) highlights that persons with different types of disability are diverse and heterogeneous, and the disability experience resulting from the interaction of health, personal and environmental factors varies significantly [4]. However, information about health needs and barriers for PWPD in China is rarely available.

It is well known that PWPD experience more restrictions on participation in social activities than people without physical disability, which is associated with lower level of well-being, including their relative poorer quality of life (QoL) [5-9]. While QoL is influenced by numerous factors [9-14], most studies have focused on demographic factors (e.g. age, gender, education, etc.) which do not account for a large proportion of variance in QoL [9]. Severity of disability, namely the activity limitation and participation restriction, has been well recognized as an objective health-related factor that influences the QoL of PWPD [15]. But even with lower degrees of severity of disability, PWPD do not necessarily have higher levels of QoL. Research has found that subjective perception and attitude on health exert substantial effect on well-being, and sometimes mediate the effect from objective health condition on QoL [16,17]. These observations can be understood based on the theoretical model of patient outcome in health-related quality of life which was proposed by Wilson [17]. According to that model, the influence of objective health condition on QoL is mediated by subjective perception on health conditions. PWPD’s own attitude towards disability is an important subjective factor, which may be associated with the severity of disability and also influences their QoL. However, most existing studies only assessed attitudes from health professionals, caregivers or the general population [11,18,19], neglecting the perspectives of PWD themselves. Although these studies suggest that others’ negative attitude significantly hamper disabled people’s QoL [20,21], the role of PWPD’s own attitude towards disability between the severity of disability and QoL remains unknown.

Besides attitude towards disability, quality of care and support (QOCS) is another important factor within the association between severity of disability and QoL among PWPD [12,22]. According to Padilla and Grant’s theoretical model on the relationship between nursing process and QoL, nursing caring and perceived caring are determinant factors on patients’ QoL [23]. On the other hand, studies have shown that the severity of disability may place greater pressure on caregivers which can influence the QOCS [24-26]. Furthermore, Chapman’s study suggests that satisfaction on caring will indirectly affect the health outcome by influencing attitude towards disability [27]. However, the role of QOCS among severity of disability, attitude and QoL has not been investigated simultaneously before.

**Figure 1:**
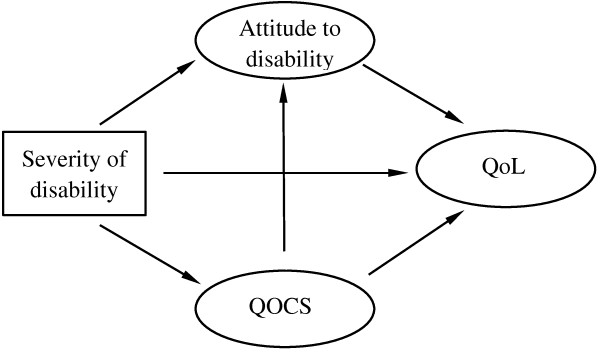
The hypothetical model of the relationship among severity of disability, attitudes to disability, quality of care and support, quality of life.

The aim of this study was to investigate the level of QoL, self-perceived QOCS, severity of disability, and personal attitude towards disability among PWPD in China, as well as to identify how QoL can be affected by severity of disability through QOCS and personal attitude towards disability among PWPD. Based on the above-reviewed literature, we hypothesized that severity of disability would have a direct relationship with attitude towards disability, QOCS and QoL. We also hypothesized that attitude towards disability and QOCS would be related to QoL, and QOCS would be related to attitude towards disability. Finally, we hypothesized that the relationship between severity of disability and QoL would be mediated by both QOCS and attitude towards disability (Figure 1).

## Methods

### Recruitment and participants

From March to August in 2008, this cross-sectional study was conducted in Guangzhou, the capital of Guangdong Province in southern China. This city has a population size of about 13 million [28] with approximately 556,000 PWD [29]. The sampling frame of this study was restricted to all PWDs who held the Disabled Person Card (DPC) in Guangzhou. Disabled Person Card, which is issued and managed by the Disabled Persons’ Federation (DPF), is PWDs’ permit to access disability benefits and allowances [30]. Participants were recruited using a multi-stage sampling technique. The first stage involved a random selection of three urban districts and two suburban districts from the total 12 districts, to ensure a proportional population distribution to that of Guangzhou, 60% of whose population resided in urban areas and 40% in suburban areas [28]. The second stage involved a random stratified selection of three sub-districts from each district, generating 15 sub-districts out of 90 sub-districts. In the final stage, cluster sampling was applied in each sub-district. Four communities per sub-district were randomly selected. Consequently, 60 communities were finally selected, and from where all residing PWPD were invited to participate in this study. Eligibility criteria included being a PWPD, a Guangzhou permanent resident, and aged 18 years or above. According to the guidelines of national DPF [31] , physical disability here is defined as “a loss of motor function of varying degrees or limitation in movements or activities resulting from deformed limbs or body paralysis or from deformity caused by damage to the structure or function of those body parts involved in mobility” [32]. The target number of PWPD from each community was 30. From a total of 1,868 eligible PWPD who were invited to participate in the study, 1,853 PWPD completed the questionnaire.

A DPF staff member and a doctor confirmed eligibility and obtained informed consent from participants. Then each participant was assigned a unique identification code for the questionnaire. The questionnaire was administered in person by an experienced research interviewer in a private room in each community. The interview was conducted in Chinese and took around 30 minutes. Each questionnaire was signed and dated by the interviewer, and all the questionnaires was reviewed for completeness and consistency by a research assistant who supervised the data collection. All the participants who completed the interview were given an assistive device (a wheelchair or a pair of crutches). The study protocol was approved by the Institutional Review Boards of Sun Yat-sen University and Guangzhou Disabled Persons’ Federation.

### Measures

#### Severity of disability scale

The 12-item World Health Organization Disability Assessment ScheduleII(WHODAS II) was used to assess the severity of disability [33]. The magnitude of disability during the previous 30 days was rated on a 5-point Likert scale (1 = none, 5 = extreme). An example item is “In the past 30 days, how much difficulty did you have in standing for a long period, such as 30 minutes?”. The average score was calculated and higher scores indicated greater severity of disability. The Cronbach’s alpha was 0.95.

### Attitude to disability scale

The Attitude to Disability Scale (ADS) was used to assess personal attitude of individuals towards their own disability [34]. The 16-item measure was scored on a 5 point Likert scale (1 = strongly disagree, 5 = strongly agree). Attitude towards disability was explained in five domains: *Inclusion* (relationships, inclusion, burden to society, burden to family), *Discrimination* (ridicule, exploitation, irritation, ignorance), *Gains* (emotional strength, maturity, achievement, determination), and *Prospects* (sexuality, underestimation, optimism, future prospects). Higher mean scores for each domain were indicative of better inclusion, less discrimination, more gains and better prospects. The Cronbach’s alphas of the domains in this study were 0.76, 0.76, 0.78, and 0.73, respectively.

### Quality of care and support (QOCS) scale

The QOCS was measured using the Quality of Care and Support Scale [35], a 17-item measure with a 5-point Likert scale (1 = not at all, 5 = totally). The QOCS Scale comprises of the following four domains: *Staff quality* (competence and knowledge of care providers, person-centered care, autonomy), *Accessibility of care* (availability of services, access to services, rights to care, cost of care), *Meeting needs* (support for leisure, social and daily living activities, standards and safety of care), and *Information* (information about disability, services, benefits, and clarity of information). Higher mean scores for each domain were indicative of higher self-perceived levels of staff quality, accessibility of care, meeting needs or information. Cronbach’s alphas were 0.78, 0.74, 0.75, and 0.83, respectively.

### Quality of Life scale for people with disability

The QoL of PWPD was measured using the short version of the WHO Quality of Life (WHOQOL-BREF) [36] and the WHO Quality of Life-Disability module (WHOQOL-DIS) [37]. The WHOQOL-BREF consists of 26 items that measure four QoL-domains: *Physical health* (pain, energy, sleep, mobility, activities, medication, work), *Psychological* (positive and negative feelings, cognitions, self-esteem, body image, spirituality), *Social relationships* (personal relationships, social support, sexual activities) and *Environmental* (safety and security, home environment, finances, health and care, information, leisure, physical environment, transport). The WHOQOL-DIS, also named *Disability* and usually used as the fifth QoL-domain for PWD, has been applied in other studies [14,37], and includes 13 items assessing specific aspects of disability. An example item is “Do you feel that other people accept you?”. All the items were scored on a 5-point Likert scale (1 = very poor, 5 = very good). Higher mean scores of each domain indicated higher levels of QoL. The Cronbach’s alphas for the five domains in this study were 0.74, 0.75, 0.58, 0.72, and 0.78, respectively.

ADS, QOCS and WHOQOL-DIS were developed by the DISQOL project: “Quality of Care and Quality of Life for People with Intellectual and Physical Disabilities: Integrated Living, Social Inclusion, and Service User Participation” [34,35,37], and this is the first application of these three cross-cultural scales among PWPD in China.

### Statistical analysis

Data were double-entered, and the two sets of data were compared using the EpiData software (EpiData 3.1 for Windows; The EpiData Association Odense, Denmark). We first performed Pearson’s correlations with SPSS 20.0 (IBM SPSS Statistics for Windows Version 20.0; IBM Corp, Armonk, NY, USA) to explore the relationship between all variables (severity of disability, QOCS, attitude to disability and QoL). Structural equation modeling (SEM) with maximum likelihood method with robust standard errors (MLR) [38] was then applied to test the whole hypothesized model by Mplus 5.0 (Mplus for Windows Version 5.0; Muthén & Muthén, Los Angeles, CA, USA). Compared with multiple linear regression, the advantage of SEM is that the whole hypothesized model can be simultaneously tested statistically, and it can handle latent variables [39]. A latent variable represents a construct that cannot be assessed directly and should be indexed with relevant indicators [40]. In this study, attitudes towards disability, QOCS, and QoL were the latent variables. The latent variable attitude towards disability was measured by four subscales: *Inclusion, Discrimination, Gains,* and *Prospects.* The latent variable QOCS was explained with four indicators: *Staff quality*, *Accessibility of care*, *Meeting needs* and *Information.* The latent variable QoL was indexed with five indicator variables: *Physical Health*, *Psychological Health*, *Social relationships, Environment* and *Disability*. Duration of disability and comorbidity, which were variables significantly associated with QOCS, attitude and QoL, were controlled as the covariates for these three latent variables in the model. The overall fit of the model was assessed with the Comparative Fit Index (CFI, ≥0.9), Tucker-Lewis Index (TLI, ≥0.9), the root mean square error of approximation (RMSEA, ≤ 0.08) [41], and the Standardized Root Mean Square Residual (SRMR, ≤ 0.08 ) [40].

Finally, direct, indirect and total effects were examined from the severity of disability to QoL. The effect of an independent variable on a dependent variable represented a direct effect, and the effect of an independent variable on a dependent variable through a mediating variable represented an indirect effect [42]. In this study, severity of disability has a direct effect on QoL, attitude towards disability and QOCS; attitude and QOCS also exert a direct effect on QoL. Meanwhile, severity of disability has the indirect effects on QoL through attitude and QOCS; QOCS also has an indirect effect on QoL through attitude. The total effect of severity of disability on QoL is the summation of the direct and indirect effects of this variable on QoL, as well as QOCS on QoL [42]. Delta method was used to examine the significance of the indirect, direct, and total effects [43].

The results reporting followed the SEM and confirmatory factor analysis (CFA) reporting guidelines which was suggested by Schreiber [44].

## Results

### Sample characteristics

Table 1 provided demographic information of the 1,853 participants. The mean age of the 1,853 participants was 51 years (range: 18-80 years old); 44.1% developed a disability before the age of five; the average duration of physical disability was 31 years (range: 0-80 years); 44.7% resided in urban areas. The mean age was similar among urban and suburban PWPD (50.4 vs. 51.2; *p* = 0.13). However, urban PWPDs reported a younger age of disability onset than suburban PWPD (11.8 vs. 25.9; *p* < 0.01). Meanwhile, urban PWPD had a higher education level (80.9% vs. 32.2% had secondary education or above, *p* < 0.01) and a higher employment rate (35.1% vs. 25.55, *p* < 0.01) than suburban ones. Of the 1,853 participants, 38.8% were comorbid with other health problems: musculoskeletal problems (arthritis, chronic back/neck pain, 30.3%), cardiovascular diseases (heart disease, hypertension, heart disease, stroke, 26.3%), respiratory problems (allergies, asthma, chronic obstructive pulmonary disease, 12.6%), neuropsychological problems (headache, dizziness, 10.5%), digestive problems (hepatitis, ulcers, 9.6%), diabetes (4.0%), sensory organ damage (hearing impairment, visual impairment, 2.3%), cancer (0.6%) and others (reproductive system, urinary system diseases, etc., 3.8%).

**Table 1:** Socio-demographic characteristics of study participants (N = 1,853)

	**n (%)**	**M (SD)**	**Range**
Gender			
Male	1125 (60.7)		
Female	728 (39.3)		
Age (years)		51.0 (12.1)	18–80
Age of disability onset		19.6 (21.3)	0–77
Marital status			
Married/cohabiting	1353 (73.0)		
Single/widowed	500 (27.0)		
Education			
Illiterate	229 (12.3)		
Primary school	624 (33.7)		
Middle /high school	963 (52.0)		
College	37 (2.0)		
Employment status			
Yes	552 (29.8)		
No	1301 (70.2)		
Residency			
Urban area	828 (44.7)		
Suburban area	1025 (55.3)		
Yearly income (CNY, 10 CNY = 1.43 USD)			
< 30,000	1470 (79.4)		
≥ 30,000	383 (20.6)		
Comorbidity			
Yes	719 (38.8)		
No	1134 (61.2)		

### Descriptive statistics

Table 2 presented correlations between all variables included in the SEM, as well as their means and standard deviations. The mean of the score on severity of disability was 1.72 (ranging from 1.00 to 5.00), indicating lower severity levels of physical disability among this study’s participants. The means of the scores on attitude (ranging from 2.75 to 3.36) and the means of the scores on the QOCS (ranging from 2.95 to 3.28) were around the midpoint, pointing towards neutral attitude towards disability and a moderate but unfavorable level of care quality. Except *Social relations* (3.22), the means of the scores on other four domains of QoL were between poor to neither poor/nor good levels (ranging from 2.65 to 2.98), indicating participants’ relatively low level of QoL.

**Table 2:** Correlation among the variables in SEM with their scale mean (M) and standard deviations (SD)

	**Mean**^**a**^	**SD**	**WHODAS**	**Attitude to disability**					**QOCS**				**QoL**			
				**INC**	**DISC**	**GAI**	**INC**	**STA**	**ACC**	**MEE**	**INF**	**PHY**	**PSY**	**SOC**	**ENV**	**DISA**
**WHODAS**	1.72	0.51	-													
**Attitude to disability**																
INC	2.75	0.54	-0.28^**^	-												
DISC	3.17	0.64	-0.25^**^	0.55^**^	-											
GAI	3.36	0.48	-0.09^**^	0.05^*^	0.00	-										
PRO	3.28	0.43	-0.17^**^	0.28^**^	0.28^**^	0.16^**^	-									
**QOCS**																
STA	3.28	0.60	-0.05^*^	0.02	0.10^**^	0.13^**^	0.10^**^	-								
ACC	3.16	0.64	-0.13^**^	0.20^**^	0.20^**^	0.00	0.14^**^	0.13^**^	-							
MEE	3.13	0.62	-0.06^**^	0.07^**^	0.06^**^	0.19^**^	0.10^**^	0.27^**^	0.04†	-						
INF	2.95	0.89	-0.32^**^	0.31^**^	0.38^**^	0.01	0.11^**^	0.22^**^	0.22^**^	0.14^**^	-					
**QoL**																
PHY	2.65	0.78	-0.58^**^	0.31^**^	0.30^**^	0.23^**^	0.17^**^	0.14^**^	0.15^**^	0.18^**^	0.26^**^	-				
PSY	2.96	0.77	-0.45^**^	0.35^**^	0.35^**^	0.24^**^	0.21^**^	0.20^**^	0.20^**^	0.21^**^	0.39^**^	0.66^**^	-			
SOC	3.22	0.63	-0.33^**^	0.24^**^	0.24^**^	0.27^**^	0.18^**^	0.25^**^	0.11^**^	0.23^**^	0.26^**^	0.47^**^	0.53^**^	-		
ENV	2.98	0.64	-0.36^**^	0.29^**^	0.27^**^	0.22^**^	0.15^**^	0.14^**^	0.11^**^	0.30^**^	0.32^**^	0.55^**^	0.60^**^	0.45^**^	-	
DISA	2.85	0.75	-0.49^**^	0.33^**^	0.30^**^	0.24^*^	0.22^**^	0.13^**^	0.18^**^	0.27^**^	0.32^**^	0.56^**^	0.59^**^	0.52^**^	0.55^**^	-

*WHODAS*, severity of disability; *INC*, inclusion; *DISC*, discrimination; GAI, gains; *PRO*: prospects; *QOCS*, quality of care and support; *STA*, staff quality; *ACC*, accessibility of care; *MEE*, meeting needs; *INF*, information; *QoL*, quality of life; *PHY*, physical health; *PSY*, psychological; *SOC*, social relations; *ENV*, environment; *DISA*, disability; †: *p* <0.10; *: *p* < 0.05; **: *p* < 0.01; ^a^:Scale ranges, 1-5.

As can be seen in Table 2, almost all correlations are significant. There were significant negative correlations between severity of disability and attitude towards disability (*r* = -0.28 to -0.09, *p* < 0.01), between severity of disability and QOCS (*r* = -0.32 to -0.05, *p* < 0.05), and between severity of disability and QoL (*r* = -0.58 to -0.33, *p* < 0.01). Significant positive correlations were found between attitude towards disability and QoL (*r* = 0.15 to 0.35, *p* < 0.01), and between QOCS and QoL (*r* = 0.11 to 0.39, *p* < 0.01). As to the correlation between QOCS and attitude towards disability, except for the relationship of *Inclusion* with *Staff quality*, *Gains* with *Accessibility*, *Information*, and *Discrimination*, all other correlation coefficients were significantly positive (*r* = 0.06 to 0.38, *p* < 0.01).

Additionally, duration of disability and comorbidity were significantly associated with QOCS, attitude and QoL. Thus, both variables were included in the following SEM as covariates.

### Results for the structural equation model

**Figure 2:**
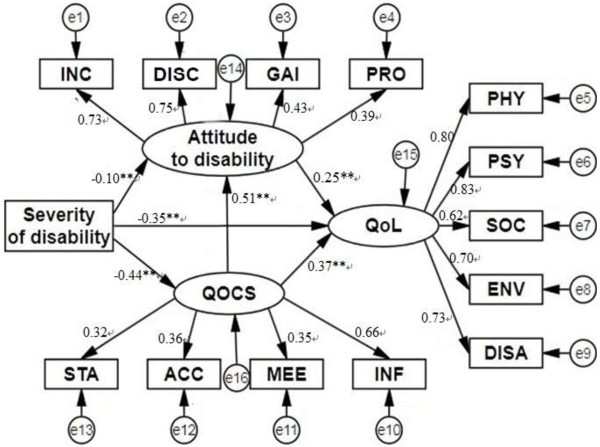
**Structural equation model examining the relationships among severity of disability, attitudes to disability, quality of care and support, quality of life among people with physical disability.** INC: Inclusion; DISC: Discrimination; GAI: Gains; PRO: Prospects; QOCS: Quality of care and support; STA: Staff quality; ACC: Accessibility of care; MEE: Meeting needs; INF: Information; QoL: Quality of life; PHY: Physical health; PSY: Psychological; SOC: Social relations; ENV: Environment; DISA: Disability; **: *p* < 0.01.

The model is presented in Figure 2. The Goodness of fit indices were: CFI = 0.91, TLI = 0.88, RMSEA = 0.061, SRMR = 0.041. PWPD’s QoL was significantly negatively influenced by severity of disability (*β* = -0.59, *p* <0.001); both QOCS (*β* = 0.50, *p* < 0.001) and attitude towards disability (*β* = 0.25, *p* < 0.001) had a positive influence on QoL among PWPD.

Table 3 presents all of the coefficients of the direct paths and summed coefficients of indirect paths in SEM. The total effect of severity of disability on QoL was -0.59 (*p* < 0.001). Out of the total effect, the direct effect from severity of disability to QoL was significant (*β* = -0.35, *p* < 0.001), which accounted for 59% of the covariance between severity of disability and QoL. Out of such total effect, the total indirect effect from severity of disability to QoL was also significant (*β* = -0.24, *p* < 0.001), and the remaining 41% of the covariance was mediated through a combination of QOCS, attitude, as well as QOCS and attitude. There were three different indirect paths that severity of disability could take to influence QoL. The first is mediated through QOCS (*β* = -0.16, *p* < 0.001), the second through attitude towards disability (*β* = -0.02, *p* < 0.05), and the third through a compound mediation of QOCS and attitude towards disability (*β* = -0.06, *p* < 0.001). The proportion of the specific indirect effect through QOCS compared to the total effect (27.4%) was much higher than through attitude towards disability (4.1%) and through the compound mediation of QOCS and attitude towards disability (9.5%) (data not shown). Similarly, the indirect effect through QOCS (*β* = -0.22, *p* < 0.001) account for 70% of the total effect (*β* = -0.32, *p* < 0.001) from severity of disability to attitude towards disability.

**Table 3:** Direct, indirect, and total effects of the model

	**QOCS**		**Attitude to disability**		**QoL**	
	***b***	***β***	***b***	***β***	***b***	***β***
Direct effects						
Severity of disability	-0.11	-0.44^**^	-0.07	-0.10^**^	-0.18	-0.35^**^
QOCS			1.55	0.51^**^	0.79	0.37^**^
Attitude to disability					0.18	0.25^**^
Indirect effects						
Severity of disability			-0.17	-0.22^**^	-0.13	-0.24^**^
QOCS					0.27	0.13^**^
Attitude to disability					-	-
Total effects						
Severity of disability	-0.11	-0.44^**^	-0.24	-0.32^**^	-0.31	-0.59^**^
QOCS			1.55	0.51^**^	1.06	0.50^**^
Attitude to disability					0.18	0.25^**^

*QOCS*, quality of care and support; *QoL*, quality of life; *b:* Regression coefficient;

*β*:Standardized regression coefficient; **: *p* < 0.01.

Overall, severity of disability accounts for 19% (*R*^*2*^ = 0.19) of variance of QOCS; severity of disability and QOCS together account for 33% (*R*^*2*^ = 0.33) of variance of attitude towards disability; severity of disability, QOCS and attitude together account for 64% (*R*^*2*^ = 0.64) of variance of QoL.

## Discussion

This study provides a snapshot of Chinese PWPD’s QoL, the severity of their physical disability, the self-perceived QOCS, and their personal attitudes towards disability. It also examines the complex association between these variables, and the findings support our hypotheses, and suggest that the influence of severity of physical disability on QoL is not only exerted directly, but also indirectly through QOCS and their personal attitudes towards disability. However, QOCS plays a more important mediating role than PWPD’s attitude towards disability.

The results indicate that, even with a mild physical disability, Chinese PWPD’s QoL, personal attitude towards disability and perceived QOCS were unfavorable, and the response usually fluctuated between “not satisfied” and “neutral”. The result of a mild physical disability is consistent with the 2006 national survey among PWD in China, which reported that 70.4% of PWD had a mild or moderate disability [45]. For the aspect of QoL, since this is one of the first studies to evaluate the level of QoL among PWD in China, we compared it with studies that also used the same *Quality of Life scale* in other countries/areas, and found that most domains of QoL in this study are worse than that of PWPD in developed countries/areas [14,46]. In general, with certain support, people with a mild disability are mostly likely to participate in the normal social life, thus achieving better wellbeing and QoL [4]. Therefore, it is meaningful to focus more on this subgroup to develop cost-effectiveness interventions to promote their participation, thus increase the average level of QoL among PWD in China. The results also showed that the worst domain of attitude towards disability was *Inclusion*. It implies that PWPD possibly experienced exclusion within Chinese society, such as difficulties in making friends, getting involved with others, as well as perceiving themselves as burdens to both their family and society [34]. This is consistent with the results of a study which showed negative public attitude towards people with disability in China [19]. A worse inclusive environment for disability is possibly because of people perceiving that disability as a kind of punishment for misdoing from a previous life in Chinese culture [47]. Therefore, people living with disability are suffering not only the disability itself, but also the stigma and discrimination which prevented them from being included in the Chinese society [48]. For the aspect of QOCS, the worst domain was *Information*, which means that PWPD in China have difficulties in accessing the information related to their rights on social services and assistance. The domains of *Accessibility of care* and *Meeting needs* were moderate, and seem somewhat better than the results from the 2006 national study among PWD in China, which reported that the percentage of unmet needs for assistance and support was more than 70% among PWD [45]. A possible explanation is that most participants of the national survey were from rural areas [45], and our sample was from both urban and suburban areas where the health service has higher quality and more accessibility. Nevertheless, this study suggests that PWPD, who live in China’s largest metropolitans, experienced a fair social inclusive environment and health care for disability, plus somewhat worse QoL, and the situation may be even worse in rural areas in China. Future studies to understand the hierarchical factors influencing social inclusion and health care delivery for PWPD are warranted in China.

Also, this study highlights the important role of QOCS within the relationship between severity of disability and QoL. It is interesting to find that the indirect pathway through QOCS accounted for one third of the total effects and over 70% of indirect effect from severity to QoL, whereas the pathway through attitude towards disability contributed less. This result suggests that even with the same level of severity, PWPD who get sufficient care and support or have a better attitude towards disability are able to achieve better QoL, but improving QOCS may be more efficient on increasing QoL than changing the attitude towards disability. Several studies proved that PWD need assistance and support to achieve a good QoL and to be able to equally participate in social life with others [4,49,50]. On the other hand, it is also well known that negative attitude is a key factor which can hamper disabled persons’ participation and inclusion in social, economic, political and culture life, consequently reducing their QoL [21]. However, there are few studies that have investigated the mediation effect of QOCS and attitude towards disability simultaneously. Improving quality of caring is especially essential for people with physical disability, since they are able to achieve relatively equal well-being if they obtain sufficient care. Also, it is obvious that improving health caring is a more specific process other than changing PWPD’s attitude, which is health workers’ priority to focus on [4]. The results of the *Quality of Care and Support scale* in this study provided an overall evaluation of the care and support that PWPD received, but the specific needs of assistance and support to promote their participation and inclusion still need to be investigated in future studies. For example, for the aspect of PWD’s caregiver quality, most assistance and support for PWD comes from their family members in China, especially in rural areas [26]. These informal caregivers have limited nursing knowledge [24,26], hence the provided caring usually may not be able to meet the needs of disabled people. But the information about these family caregivers of PWD is limited in China, such as their health care quality, courtesy stigma, their attitudes towards disability, their physical and mental burdens, and their QoL, etc. China governments and public service organizations have placed higher priority on improving the lives of persons with disabilities, providing more services to ensure “Equality, Participation and Sharing” of PWD, from several different aspects, including education, rehabilitation, employment, social security, and social environment, etc [51]. However, the knowledgement and the accessibility of these services are far from optimistic. In China, only 35.6% PWD had ever received medical services and aid; 12.5% had ever received aid and support services; 8.5% had ever received rehabilitation and training services; and only 7.3% had ever received free assistive devices [45]. More research is warranted to better understand the current problems and barriers to achieving sufficient caring and support for disabled persons, and what works in overcoming them in different contexts, such as under different kinds of disability, rural or urban setting, etc.

It is also worthwhile to notice that, most PWPD in this study had lower level education, were currently unemployed, and were poorer than average level. In China, around two fifths of PWD who were over 15 years old were illiterate, and 85% of poor PWD had never advanced beyond middle school education [45]. There was no schooling available for children with disabilities before 1979 in China. After Compulsory Education Law passed in 1986, Law of the People’s Republic of China on the Protection of Disabled Persons finally allowed children with disabilities equal rights to children without disabilities to access nine years of education, including six years elementary and three years middle school education [52]. The average age of our sample was 51 years old, which means most of them were not able to access the universal education at their school age. A lower education level may affect employment, which in turn might lead to their lower economic status in society. That is possibly the reason why most of the participants were currently unemployed and poor. However, no empirical evidence exists in China to link this potential socioeconomic relationship. Longitudinal studies are needed to establish the causal relation between disability, education, employment, and poverty under different context for youth generation with disability in China. Furthermore, the education level of PWPD from suburban areas was significantly lower than those from urban areas. The lower education level thus led to suburban PWPD’s significant lower employment rate. Further studies are warranted to identify the differences in the accessibility and equality of all opportunities among urban, suburban and rural PWD in China. These results also reflected unfavorable conditions of the implementation on the policy of disabled persons’ equal rights to education in suburban or rural China. The translation of disability-related policy implementation from urban context to suburban or rural context also needs to be further investigated in China, especially for coordination of local resources and personnel to achieve the equal rights for PWD in rural areas [4].

The study’s results should be viewed in light of some limitations. The present study used a cross-sectional design so that causal relationships cannot be drawn. Furthermore, the study sample was heterogeneous in etiology, however, this limitation has been shared by other published studies [20,53]. Finally, among this sample, many participants reported a longer duration of years of disability which may have adapted them to their disability, thus their attitude towards disability and their demands or needs may be quite different from people experiencing a newer disability. However, we adjusted the duration of disability in our model to address this issue, but further longitudinal studies are warranted to uncover the potentially different trends in attitude and access to services depending on length of time with disability.

## Conclusions

Despite these limitations, the results of this study evaluate the health status of PWPD in China, and also yield important insights into how the severity of disability, QOCS and attitude towards disability influence the QoL of PWPD. This study inferred that focusing on improvement of assistance and care services has the potential to substantially improve PWPD’s QoL. It also inferred that further research should focus on understanding the needs and care services accessibility of PWPD in different areas of China, thus being able to develop better interventions and implement services for them.

## Abbreviations

PWPD: People with physical disability; PWD: People with disability; WHO: World Health Organization; QoL: Quality of life; QOCS: Quality of care and support; DPC: Disabled person card; DPF: Disabled persons’ federation; SEM: Structural equation modeling; CFI: Comparative fit index; TLI: Tucker-lewis index; RMSEA: Root mean square error of approximation; SRMR: Standardized root mean square residual.

## Competing interests

The authors declare that they have no competing interests.

## Authors’ contributions

All authors contributed to this work. Most of the authors contributed to the study design and protocol development (QZ, QT, JG, ZL, XC, JF, JR, QA, YH). QZ also contributed to the data collection and data analysis. Furthermore, QZ and QT contributed to the results interpretation and writing of the manuscript. JG and R L-C also contributed to the writing and revising of the manuscript. CH contributed to the writing, reviewing, and final editing of the manuscript. YH not only contributed to the study design and manuscript revision, but also supervised the project progress. CH and YH takes full responsibility for the integrity of the data and the accuracy of the data analysis. All authors read and approved the final manuscript.
